# Targeted regulation of fibroblast state by CRISPR-mediated CEBPA expression

**DOI:** 10.1186/s12931-019-1253-1

**Published:** 2019-12-11

**Authors:** Wei Liu, Jeffrey A. Meridew, Aja Aravamudhan, Giovanni Ligresti, Daniel J. Tschumperlin, Qi Tan

**Affiliations:** 1 Emergency Department, First Hospital of China Medical University, Shenyang, China; 2 Department of Physiology & Biomedical Engineering, Mayo Clinic, Rochester, Mayo Clinic College of Medicine and Science, 200 1st St SW, Rochester, MN 55905 USA; 3 Department of Medicine, Boston University School of Medicine, Boston, MA USA

**Keywords:** CEBPA, Lung fibrosis, Fibroblast activation, Adipogenesis, Lipofibroblast, CRISPR activation

## Abstract

**Background:**

Fibroblasts regulate tissue homeostasis and the balance between tissue repair and fibrosis. CCAAT/enhancer-binding protein alpha (CEBPA) is a key transcription factor that regulates adipogenesis. CEBPA has been shown to be essential for lung maturation, and deficiency of CEBPA expression leads to abnormal lung architecture. However, its specific role in lung fibroblast regulation and fibrosis has not yet been elucidated.

**Methods:**

Lung fibroblast CEBPA expression, pro-fibrotic and lipofibroblast gene expression were assessed by qRT-PCR. CEBPA gain and loss of function experiments were carried out to evaluate the role of CEBPA in human lung fibroblast activation with and without TGF-β1 treatment. Adipogenesis assay was used to measure the adiopogenic potential of lung fibroblasts. Finally, CRISPR activation system was used to enhance endogenous CEBPA expression.

**Results:**

We found that CEBPA gene expression is significantly decreased in IPF-derived fibroblasts compared to normal lung fibroblasts. CEBPA knockdown in normal human lung fibroblasts enhanced fibroblast pro-fibrotic activation and ECM production. CEBPA over-expression by transient transfection in IPF-derived fibroblasts significantly reduced pro-fibrotic gene expression, ECM deposition and αSMA expression and promoted the formation of lipid droplets measured by Oil Red O staining and increased lipofibroblast gene expression. Inhibition of the histone methyl transferase G9a enhanced CEBPA expression, and the anti-fibrotic effects of G9a inhibition were partially mediated by CEBPA expression. Finally, targeted CRISPR-mediated activation of CEBPA resulted in fibroblasts switching from fibrogenic to lipofibroblast states.

**Conclusions:**

CEBPA expression is reduced in human IPF fibroblasts and its deficiency reduces adipogenic potential and promotes fibrogenic activation. CEBPA expression can be rescued via an inhibitor of epigenetic repression or by targeted CRISPR activation, leading to reduced fibrogenic activation.

## Background

Idiopathic pulmonary fibrosis (IPF) is a chronic progressive fibrotic disease of unknown etiology that is marked by epithelial cell injury, progressive myofibroblast activation, aberrant deposition of extracellular matrix proteins, ultimately resulting in failure of the respiratory system and death [1]. It is widely thought that the pathogenesis of IPF starts in the alveolar region of the lung, leading to the emergence of active pro-fibrotic fibroblasts located in fibrotic foci [2]. Recent lineage tracing studies indicate that lipofibroblasts may differentiate into activated myofibroblasts following tissue injury to promote ECM remodeling [3, 4].

Pulmonary lipofibroblasts are located adjacent to alveolar epithelial type II cells (AEC2) in the distal lung, and are recognized by the presence of lipid droplets [5]. Lipofibroblasts are well characterized in rodent lungs, while their presence in human lungs is more controversial [6]. Lipofibroblasts are implicated in alveolar maturation and surfactant production [7], as well as epithelial mesenchymal interactions and homeostasis in the lung [8, 9], thus their presence or absence may play important roles in chronic lung diseases [10]. The mechanisms leading to fibroblast phenotype changes in diseased lung fibroblasts and the functional implications on their fibrogenic activation are not fully understood.

CCAAT enhancer-binding protein alpha (CEBPA) is a member of the basic leucine zipper (bZIP) family of transcription factors. CEBPA interacts with CDK2 and CDK4 to play important roles limiting proliferation [11, 12]. CEBPA also plays a key role in regulation of adipogenesis [13] and has been proposed to contribute to lipofibroblast fate acquisition [14] by activating adipose-specific genes, though direct evidence in support of this remains lacking. Single-cell sequencing of fibroblasts in both normal adult and fibrotic mouse lung has revealed heterogeneous fibroblasts subtypes including myofibroblasts and lipofibroblasts, and recent data suggest that CEBPA is repressed in the lung during bleomycin induce fibrosis [14, 15], accompanying a shift in fibroblast state from lipo- to myofibroblast.

Alterations in whole lung CEBPA gene expression have previously been linked to altered lung development and chronic lung diseases [16–18]. Lung-specific inactivation of CEBPA impairs lung development and epithelial differentiation, with animals developing a severe pathological state similar to chronic obstructive pulmonary disease in a mice model [19]. However, the expression of CEBPA in healthy and pathologically activated lung fibroblasts is less understood, and its roles in controlling fibroblast activation and homeostasis have not yet been extensively investigated.

Here, we aimed to establish CEBPA as a critical modulator that promotes the lipofibroblast phenotype while reducing fibrogenic cell activation, and identify epigenetic and targeted mechanisms by which CEBPA expression can be restored.

## Methods

### Cell culture and adipogenesis assay

Primary human lung fibroblasts isolated by explant culture from the lungs of subjects diagnosed with IPF who underwent lung transplantation, or donors whose organs were rejected for transplantation (non-IPF) were kindly provided by Peter Bitterman and Craig Henke at the University of Minnesota under a protocol approved by the University of Minnesota Institutional Review Board and by Carol Feghali-Bostwick at the Medical University of South Carolina under a protocol approved by the University of Pittsburgh Institutional Review Board. Primary human lung fibroblasts (HLFs) were used between passages 3 and 7. HLFs were maintained in DMEM supplemented with 10% FBS. All cells were regularly maintained at 37 °C in a humidified, 5% CO2 atmosphere. The culture medium was changed every other day.

For adipogenesis assay, HLFs were cultured in DMEM supplemented with 10% FBS. Upon confluency, cells were switched to an adipogenic differentiation cocktail: DMEM supplemented with 1% ITS premix (insulin-transferrin-selenium; Thermo Fisher Scientific, 41400045), 0.5 mM isobutylmethylxanthine (Sigma, I5879), 0.1 mM cortisol (StemCell Techonologies, 07925), 1 mM dexamethasone (Sigma, D4902), 0.2 nM triiodothyronine (Sigma, T6397), and 1 mM rosiglitazone (Cayman Chemical, 71740). Cells were maintained in the induction media for 7 days and the medium was changed every other day. At Day 7–10, Lipid droplets were identified by Oil Red O staining. Cells were fixed in 10% formalin (Sigma-Aldrich) for 1 h at 4 °C and stained with a 0.5% solution of Oil-Red-O (Sigma-Aldrich, O1516) in 60% isopropanol for 15 min at room temperature. Oil Red O staining was measured semi-quantitatively by extracting Oil Red O stain with 100% isopropanol for 10 min gentle rocking and reading absorbance at 492 nm.

### RNA interference

Transient RNA interference was performed with SMARTpool: ON-TARGETplus CEBPA siRNA (L-006422-00-0005, Dharmacon, Lafayette, CO, USA) or with On-TARGETplus Nontargeting Pool (D-001810-10-05, Dharmacon, Lafayette, CO, USA) as control by using Lipofectamine RNAiMAX reagent (Thermo Fisher Scientific, 13778075) following manufacturer’s protocols. Cells were harvested after 48–72 h for RNA and protein analysis.

### Plasmids and transfection

pScalps_Puro_mCebpa is an expression plasmid of C/EBPα and was originally deposited by Silvia Monticelli [20]. Transient transfection was performed with pScalps_Puro_mCebpa (79551, Addgene, Cambridge, MA, USA) or control plasmid using Lipofectamine 3000 reagent (Thermo Fisher Scientific, L3000015) according to manufacturer’s protocols. Expression of the constructs in the transfections was determined by qRT-PCR and Western blot.

### CRISPR activation

In this study, dCas9-VPR plasmids were utilized to generate cells expressing dCas9-VPR. SP-dCas9-VPR was originally generated by George Church (Addgene plasmid # 63798; http://n2t.net/addgene:63798; RRID:Addgene_63798) [21]. Transient transfection was performed with SP-dCas9-VPR plasmid using Lipofectamine 3000 reagent (Thermo Fisher Scientific, Waltham, MA, USA) according to manufacturer’s protocols. Expression of the dCas9 was confirmed by Western blot. These cells were then transfected by individual synthetic Edit-R CRISPRa Human CEBPA crRNA (CA-006422-01-0002, CA-006422-02-0002, CA-006422-03-0002, CA-006422-04-0002, Dharmacon, Lafayette, CO, USA) and Edit-R CRISPR-Cas9 Synthetic tracrRNA (U-002005-05, Dharmacon, Lafayette, CO, USA), which was carried out by lipid transfection at the recommended crRNA:tracrRNA working concentration (25 nM:25 nM). After 3–5 days, RT-qPCR was used to confirm target gene activation.

### RNA extraction and qRT-PCR analysis

Total RNA was isolated with RNeasy Plus Mini kit. cDNA was synthesized with SuperScript™ IV Reverse Transcriptase. qRT-PCR was carried out using the FastStart Essential DNA Green Master (Roche, 06402712001) for SYBR Green I-based real-time PCR on the Lightcycler 96 Real-Time PCR System (Roche) according to the manufacturer’s instructions. qRT-PCR was performed by incubating the plates at 95 °C for 10 min and then cycling 40 times at 95 °C for 10 s, 60 °C for 10s, and 72 °C for 10s. Ct values within each experiment were normalized against GAPDH, and fold change calculated for all conditions relative to a single, randomly selected, control result. The primers were designed to be human gene-specific and are listed in Table 1.

**Table 1:** Primers for qRT-PCR analysis are listed. “CEBPA” primers were used to measure human fibroblast endogenous gene expression, and “Cebpa” primers used to detect the expression of pScalps_Puro_mCebpa plasmid

gene names	Sequences
GAPDH-F	AATGAAGGGGTCATTGATGG
GAPDH-R	AAGGTGAAGGTCGGAGTCAA
CEBPA-F	GCAAACTCACCGCTCCAATG
CEBPA-R	GGAAGGAGGCAGGAAACCTC
ACTA2-F	GTGTTGCCCCTGAAGAGCAT
ACTA2-R	GCTGGGACATTGAAAGTCTCA
COL1Α1-F	GAGGGCCAAGACGAAGACATC
COL1Α1-R	CAGATCACGTCATCGCACAAC
FN1-F	AGGAAGCCGAGGTTTTAACTG
FN1-R	AGGACGCTCATAAGTGTCACC
CTGF-F	CAGCATGGACGTTCGTCTG
CTGF-R	AACCACGGTTTGGTCCTTGG
PLIN2-F	ATGGCAGAGAACGGTGTGAAG
PLIN2-R	CAACTGCAATTTGCGGCTC
PPARA-F	GCTTTCTGGGTGGACTCAAGT
PPARA-R	GAGGGCAATCCGTCTTCATCC
PPARG1-F	GGGATCAGCTCCGTGGATCT
PPARG1-R	TGCACTTTGGTACTCTTGAAGTT
PPARGC1A-F	GCTTTCTGGGTGGACTCAAGT
PPARGC1A-R	GAGGGCAATCCGTCTTCATCC
Cebpa-f	CAAGAACAGCAACGAGTACCG
Cebpa-r	GTCACTGGTCAACTCCAGCAC

### Immunofluorescence microscopy and image quantification

The cells were fixed in 4% paraformaldehyde and permeabilized in 0.25% Triton X-100 (Sigma-Aldrich, St. Louis, MA, USA). After blocked with 1% BSA for 1 h, the cells were incubated with anti-αSMA (F3777, Sigma-Aldrich, St. Louis, MA, USA) conjugated antibody (diluted 1:200 in PBS with 1% BSA) overnight at 4 °C, followed by DAPI to counterstain nuclei. The images were automatically captured and quantified by Cytation 5 Imaging Reader (BioTek).

### Western blot assay

Cells were harvested into RIPA Lysis Buffer (Thermo Fisher Scientific, 89900) with Halt Protease and Phosphatase Inhibitor Cocktail (Thermo Fisher Scientific, 78440). Lysates were then quantitated using Pierce BCA Protein Assay Kit (Thermo Fisher Scientific, 23225); and equal amounts of protein were subjected to 4–15% Mini-PROTEAN® TGX™ Gel (Bio-Rad, 15 μl #4568086 or 50 μl #4561084). Proteins were then transferred from the gel to PVDF membranes with Trans-Blot Turbo Transfer System (Bio-Rad). After Blocking for 1 h at room temperature with 5% nonfat dry milk (Bio-Rad, Bloting-Grade Blocker, 1706404) in TBST blocking buffer, PVDF membranes were probed with GAPDH (Cell Signaling Technology, 2218S), CEBPA (Cell Signaling Technology, 8178S), α-SMA (Sigma, F3777), Fibronectin (Santa Cruz Biotechnology, sc-9068), Smad2/3 (Cell Signaling Technology, 8685S) and pSmad2/3 (Cell Signaling Technology, 8828S) antibodies at 4 °C overnight followed by incubation with HRP conjugated goat anti-rabbit (Promega, W4011) or anti-mouse IgG (Promega, W4021) for 1 h at room temperature. Bands were detected by using Super Signal West Pico Plus (Thermo Fisher Scientific, 34580) and visualized using a BioRad ChemiDoc Imaging system (Bio-Rad). The quantification was performed via densitometry with the expression of specific antibody relative to GAPDH was computed in the ImageLab software provided by Bio-Rad. Data are expressed as normalized with experiment control for each independent experiment.

### Immuno-ECM assay

The cells were plated at 5X10^4^ cells per well in 96 well plates until 70–90% confluence for treatment. After 48 h treatment, the cells were ready for the immune-ECM assay. The culture media was gently removed from the samples, followed by two washes with PBS. Samples were fixed in 4% paraformaldehyde for 15 min. After two additional PBS washes, cells were treated with Odyssey Blocking Buffer for 45 min. Cells were incubated with primary polyclonal rabbit antibody for collagen I (Novus NB600–408) or fibronectin (Santa Cruz, sc-81767) diluted 1:200 in blocking buffer at 4 °C overnight. Cells were washed twice in PBS and incubated with 100uL of the corresponding secondary antibodies solution, which were prepared from IRDye® 800CW (Li-Cor, 926–32211) and IRDye® 680CW (Li-Cor, 926–68070) secondary antibodies in blocking buffer solution at a 1:750 ratio. The plate was imaged on the Li-Cor Odyssey system. Plates were imaged via a Li-Cor OdysseyXL system with quantification performed via densitometry and normalized with cell density. Data are expressed as IR intensity fold changes relative to control.

### Statistical analysis

All results are expressed as mean ± SD. Comparison between two groups were performed using non-parametric Mann-Whitney test and comparison of more than two groups were analyzed with One-way ANOVA. GraphPad Prism 8 was used to perform these statistical analyses (GraphPad Software, San Diego CA, USA). *P* values of < 0.05 were considered statistically significant. All experiments were repeated at least three times, and representative data are shown.

## Results

### Loss of CEBPA in normal lung fibroblasts enhanced ECM deposition

**Fig. 1:**
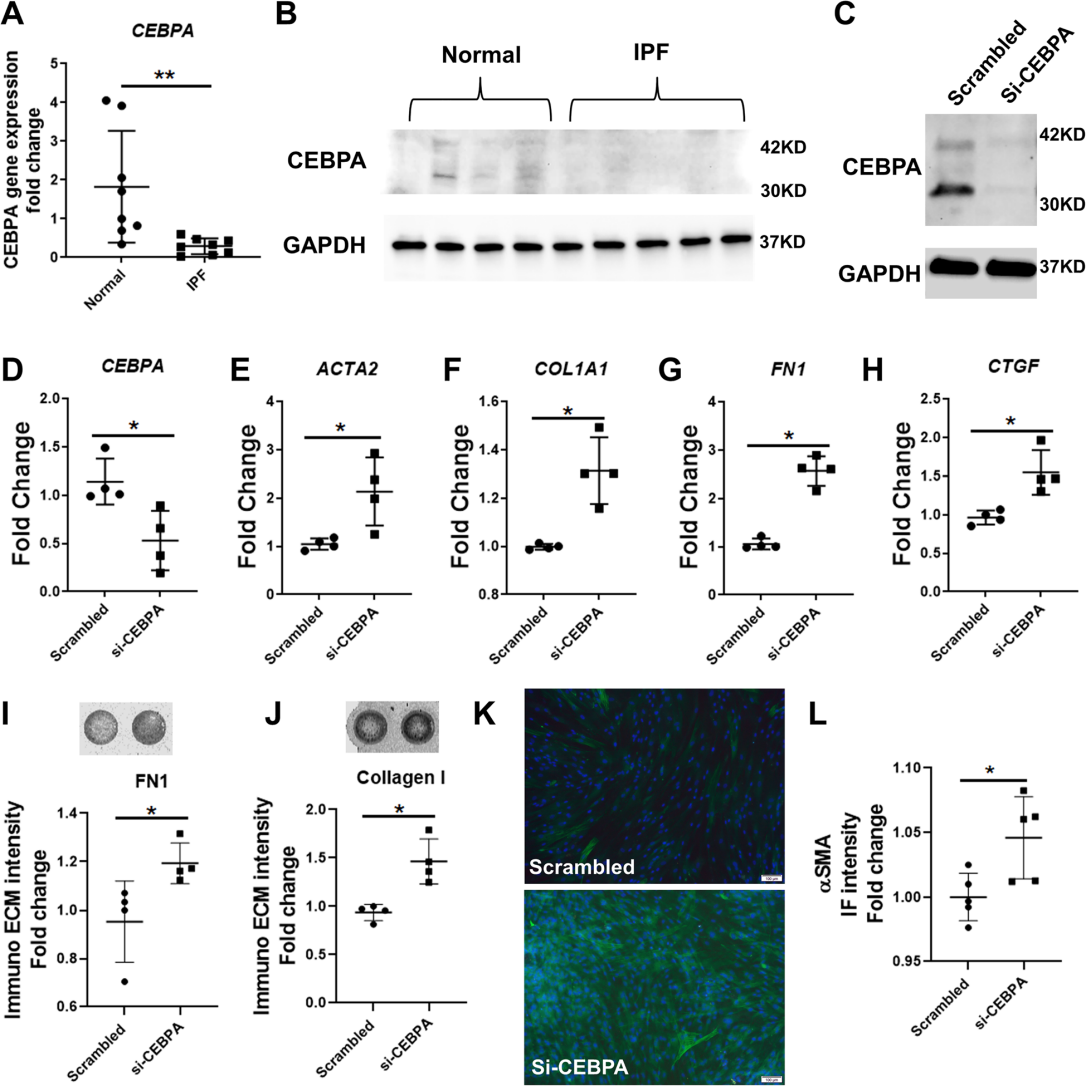
Loss of CEBPA in normal lung fibroblasts enhanced ECM deposition. **a**) qRT-PCR analysis showing *CEBPA* expression in IPF-derived fibroblasts (*N* = 8) and healthy fibroblasts (N = 8). **b**) Western blotting analysis showing CEBPA protein expression in IPF-derived fibroblasts (*N* = 5) and healthy fibroblasts (*N* = 4). **c**) Western blotting analysis and **d**) qRT-PCR confirms the knock down of CEBPA expression. **e-h**) qRT-PCR analysis showing *ACTA2*, *COL1A1*, *FN1* and *CTGF* transcript levels in CEBPA knock down normal fibroblasts and their control. **i-j**) ECM deposition assay shows collagen I and fibronectin production in CEBPA knockdown lung fibroblasts and their control. **k**) Immunostaining and l) quantification for αSMA expression in both CEBPA knockdown lung fibroblasts and control lung fibroblasts. Scale bar = 100 μm. Data are expressed as mean ± SD (*p < 0.05, ** *p* < 0.01)

To directly characterize human-disease relevant changes in CEBPA expression, we compared human IPF-derived and healthy control lung fibroblasts. Across donors we observed a significant decrease in *CEBPA* transcript levels between fibroblasts derived from lungs of healthy donors versus those derived from subjects with IPF (Fig. 1a). Western blotting confirmed these changes, with modest and variable CEBPA protein expression in healthy control fibroblasts and undetectable CEBPA protein levels in IPF fibroblasts (Fig. 1b).

To test whether reduced expression of CEBPA in normal lung fibroblasts directly alters their fibrogenic responses, we used CEBPA siRNA to knock down CEBPA in primary normal human lung fibroblasts isolated from healthy donors (HLF), as confirmed by qRT-CPR (Fig. 1d). Western blotting confirmed knock down of CEBPA protein (Fig. 1c) in the HLF, and qRT-PCR demonstrated that CEBPA knockdown leads to increased expression of transcripts for pro-fibrotic genes ACTA2, COL1A1, FN1 and CTGF (Fig. 1e-h). Immuno-ECM assay revealed that deposition of fibronectin (Fig. 1I) and type 1 collagen (Fig. 1j) were significantly increased in HLF with CEBPA knockdown. From immunofluorescence staining, we observed αSMA staining increased (Fig. 1k, l) as well as cell number increased (counted by DAPI, Additional file1: Figure S1A) in HLF with CEBPA knockdown as well.

### CEBPA overexpression in IPF-derived fibroblasts reduces their fibrogenic activation

**Fig. 2:**
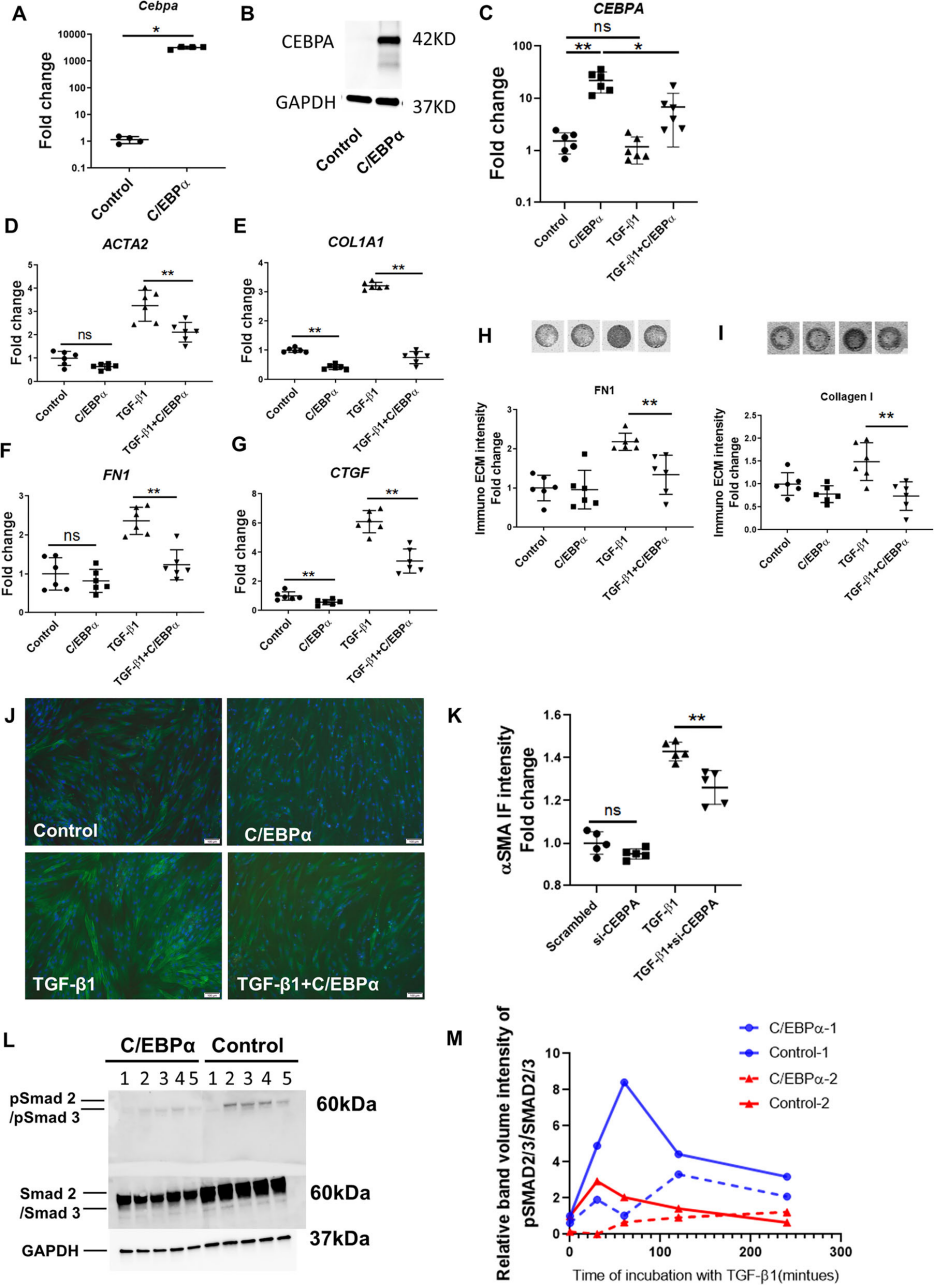
CEBPA overexpression in IPF-derived fibroblasts reduces their fibrogenic activation. **a**) qRT-PCR analysis of Cebpa expression in the C/EBPα plasmid-overexpressing IPF fibroblasts compared to empty vector transfected control. **b**) Western blotting analysis of C/EBPα protein in the C/EBPα-overexpressing IPF fibroblasts compared and empty vector transfected control. **c-g**) qRT-PCR analysis showing *CEBPA*, *ACTA2*, *COL1A1*, *FN1* and *CTGF* transcript levels in the C/EBPα-overexpressing IPF fibroblasts compared and empty vector transfected control. **h-i**) ECM deposition assay shows collagen I and fibronectin production in the C/EBPα-overexpressing IPF fibroblasts compared to empty vector transfected control. **j-k**) Immunostaining for αSMA expression in the C/EBPα-overexpressing IPF fibroblasts compared to empty vector transfected control. Scale bar = 100 μm. l) Western blot analysis of phospho-SMAD2/3 (pSMAD2/3) and total SMAD2/3 and GAPDH protein expression in the C/EBPα-overexpressing IPF fibroblasts and empty vector transfected control with TGF-β for the indicated periods. Time point 1–5: 0 min, 30 min, 60 min, 120 min, 240 min. m) Quantification of the pSMAD2/3 expression to total SMAD2/3 ratio from two independent experiment. Data are expressed as mean ± SD (*p < 0.05, ** *p* < 0.01)

To test whether enhanced expression of CEBPA can reverse the pro-fibrotic fibroblast state, we transfected IPF-derived fibroblasts with a plasmid expressing C/ebpα protein (C/ebpα refers to exogenous plasmid in the all figures). After 48 h, over expression of CEBPA alone in IPF derived fibroblasts significantly increased Cebpa transcript (Fig. 2a), endogenous CEBPA transcript (Fig. 2c) and CEBPA protein levels (Fig. 2b), and reduced ACTA2, COL1A1, FN1 and CTGF gene expression (Fig. 2d-g). To determine whether CEBPA attenuates the pro-fibrotic effect induced by TGF-β1, cells were treated with 5 ng/ml TGF-β1 for 48 h and we observed the expression of pro-fibrotic genes ACTA2, COL1A1, FN1, CTGF (Fig. 2d-g) and SERPINE1 (Additional file 1: Figure S1B) were reduced significantly in response to CEBPA over expression compared to control TGF-1 treated cells. Reductions were also detected in αSMA immunofluorescence staining (Fig. 2l, k) and deposition of fibronectin (Fig. 2h) and type 1 collagen (Fig. 2I) in cells with CEBPA overexpression compared to control. We did not observe any changes in expression of cellular senescence markers CDKN2A and GLB1 due to CEBPA overexpression or TGF-β1 treatment (Additional file 1: Figure S1C, D).

TGF-β1 induced pro-fibrotic effects are mediated in part by SMAD2/3 signaling. We thus investigated whether CEBPA expression altered the magnitude of the SMAD2/3 phosphorylation response to TGF-β1. IPF fibroblasts were transfected with CEBPA expression plasmid and 48 h later TGF-β1 (5 ng/ml) was added into the medium and protein was collected at different time points after the addition of TGF-β1. Western blot results showed that both SMAD2/3 phosphorylation and total SMAD2/3 was reduced in CEBPA overexpression groups compared to control groups (Fig. 2l). The quantified results showed CEBPA overexpression reduced the ratio of SMAD2/3 phosphorylation to total SMAD2/3 induced by TGF-β1 (Fig. 2m). Together these results demonstrate that over-expression of CEBPA alone is sufficient to reduce fibrogenic fibroblast activation at baseline and in response to TGF-β1.

### CEBPA expression promotes a lipofibroblast phenotype

**Fig. 3:**
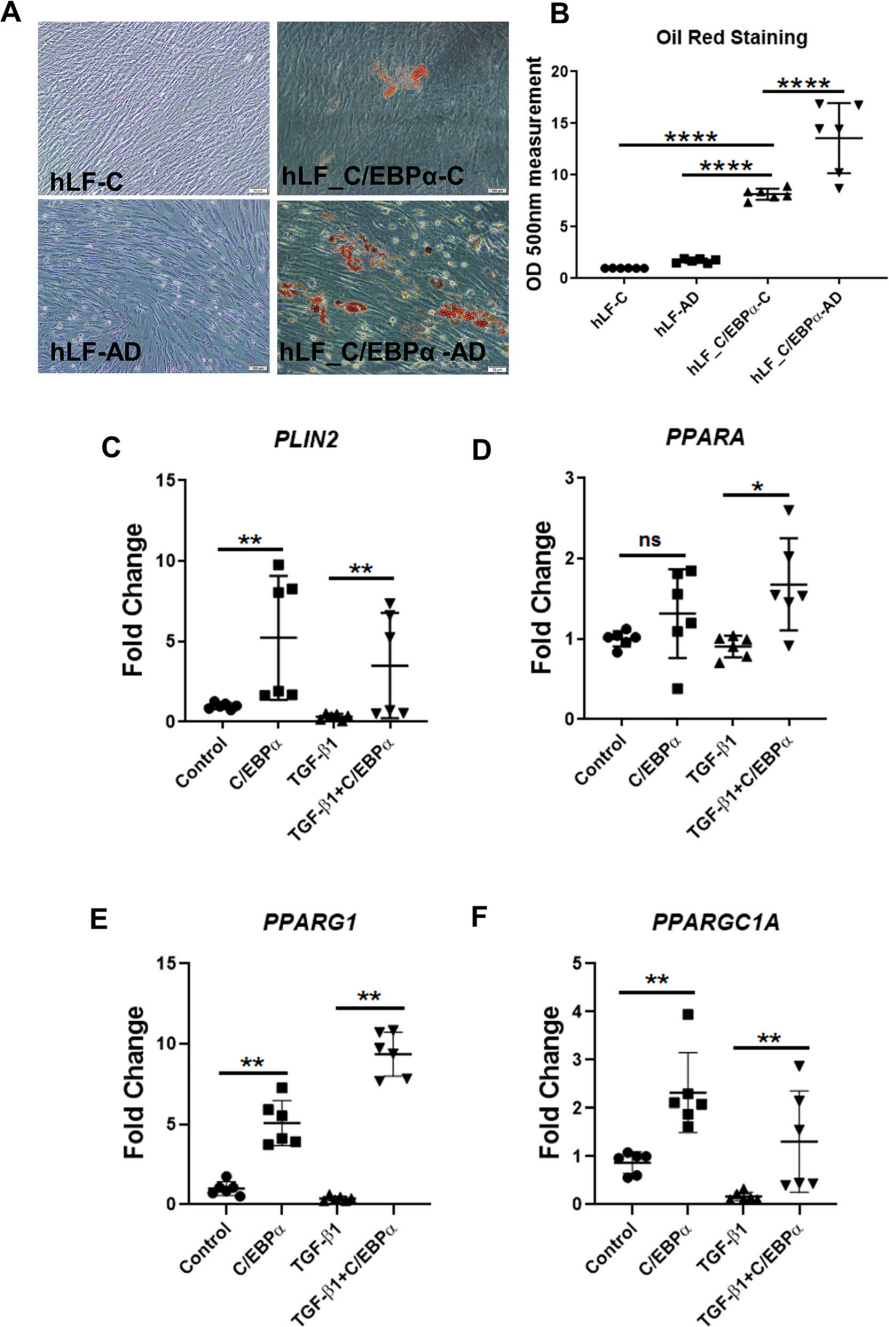
CEBPA expression promotes a lipofibroblast phenotype.**a-b**) Oil Red O staining and quantification in the Cebpa-overexpressing IPF fibroblasts and empty vector transfected control with (AD: adipogenic medium) or without adipogenic medium induction (**c**: control medium). Scale bar = 100 μm. **c-f**) qRT-PCR analysis of *PLIN2*, *PPARA*, *PPARG1* and *PPARGC1A* transcript levels in Cebpa-overexpressing IPF fibroblasts and empty vector transfected control with or without TGF-β1 treatment for 48 h. Data are expressed as mean ± SD (*p < 0.05, ** *p* < 0.01, ***p < 0.001, **** *p* < 0.0001)

Based on evidence implicating fibroblast fate switching between lipogenic and myogenic states in lung fibrosis and resolution [3], and CEBPA in lipogenesis [13], we hypothesized that CEBPA expression in lung fibroblasts regulates the lipofibroblast phenotype. To test this concept, we first examined adipogenic differentiation of fibroblasts using CEBPA overexpression or an adipogenic cocktail [4]. Oil Red O staining showed that CEBPA transfection alone was able to promote the formation of oil droplets that were not present in untreated controls (Fig. 3a). Adipogenic medium alone was unable to promote oil droplet formation, but did significantly enhance the effect of CEBPA expression on the adipogenic response of human lung fibroblasts (Fig. 3a, b). CEBPA overexpression increased the transcripts for lipofibroblast genes *PLIN2, PPARA*, *PPARG1* (Fig. 3c-e) as well as the PPAR co-activator *PPARGC1A* (encoding PGC1α) (Fig. 3f), both at baseline and in the presence of TGF-β1. Collectively, these results demonstrate that enhancing CEBPA with ectopic expression in IPF-derived fibroblasts increases adipogenesis potential and lipofibroblast marker expression.

### CEBPA expression is enhanced by a G9a inhibitor and partially mediates its anti-fibrotic effects

**Fig. 4:**
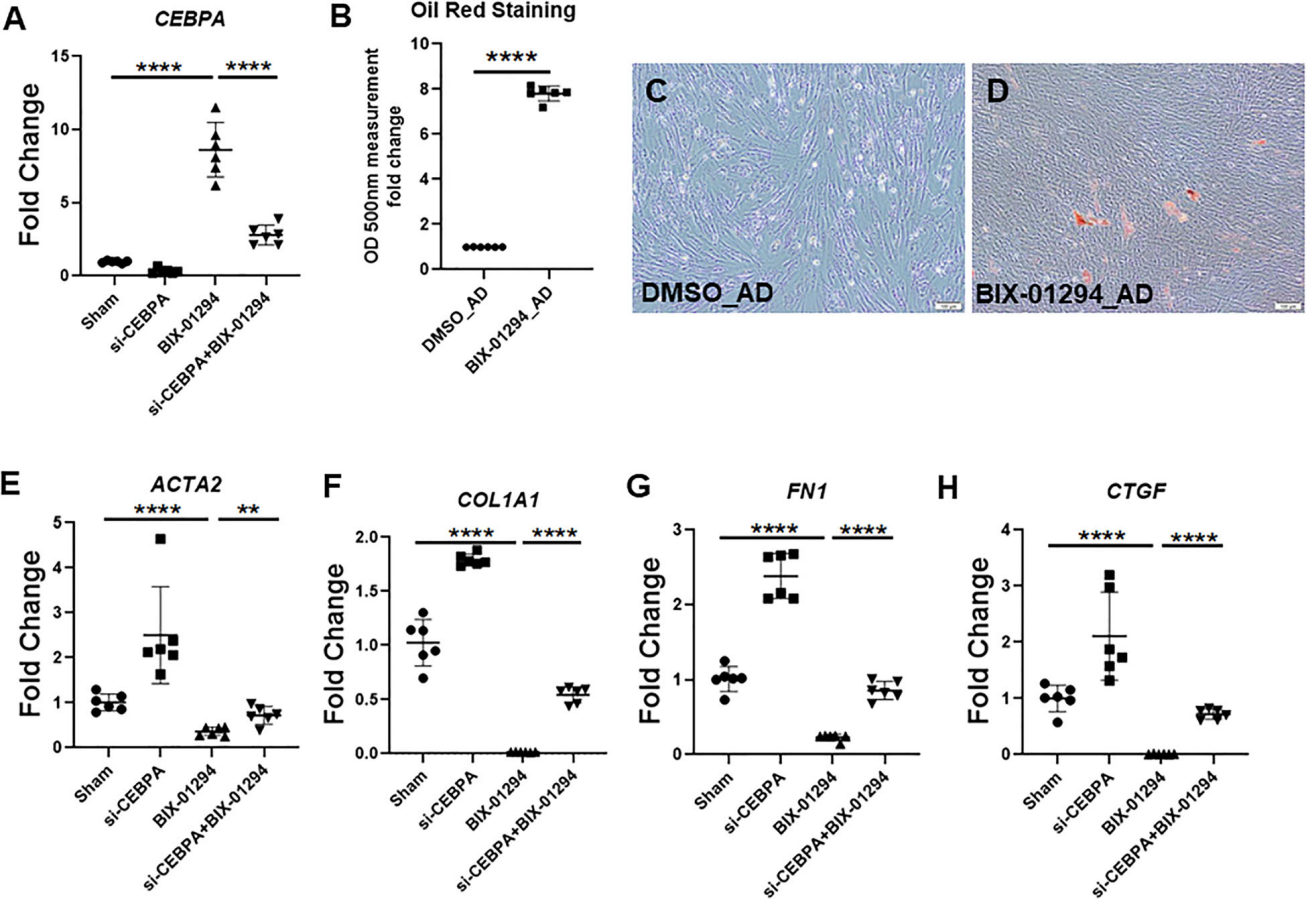
CEBPA expression is enhanced by a G9a inhibitor and partially mediates its anti-fibrotic effects. **a**) qRT-PCR analysis of *CEBPA* expression in CEBPA knock down lung fibroblasts and their control with or without BIX01294 for 48 h. **b-d**) Oil Red O staining and their quantification in IPF fibroblasts with or without BIX01294 in the adipogenic medium for 10 days. **e-h**) qRT-PCR analysis showing *ACTA2*, *COL1A1*, *FN1* and *CTGF* transcript levels in CEBPA knock down lung fibroblasts and their control with or without BIX01294 for 48 h. Data are expressed as mean ± SD (*p < 0.05, ** *p* < 0.01, ***p < 0.001, **** *p* < 0.0001)

Our findings of CEBPA repression in IPF lung fibroblasts and the beneficial effects of restoring its expression on attenuating pro-fibrotic fibroblast activation (Fig. 2) suggest that pharmacological rescue of CEBPA expression may have therapeutic relevance. Previous work has shown that the epigenetic complex G9a/CBX5 represses expression of genes that maintain or restore the quiescent state of lung fibroblasts, and inhibition of G9a can reverse IPF fibroblast activation and experimental lung fibrosis [22]. To test whether G9a inhibition has similar effects on CEBPA in human IPF-derived lung fibroblasts, we exposed cells to a G9a inhibitor, BIX01294 using a dose effective in previous study [22]. Treatment of IPF fibroblasts with BIX01294 potently upregulated *CEBPA* expression (Fig. 4a) and reduced the transcripts for pro-fibrotic genes *ACTA2, COL1A1, FN1*, and *CTGF* in these cells (Fig. 4e-h). To evaluate the effect of BIX01294 on the lipofibroblast phenotype, BIX01294 was combined with adipogenic medium for 10 days. Oil Red O staining demonstrated BIX01294 treatment restored the adipogenic potential of IPF fibroblasts (Fig. 4b-d).

To determine the involvement of CEBPA in mediating the anti-fibrotic effects of BIX01294, we transfected *CEBPA* siRNA or non-targeting control siRNA into IPF fibroblasts in presence of BIX01294 for 48 h. *CEBPA* siRNA significantly reduced the expression *CEBPA* in the BIX01294 treatment group. Knock down of CEBPA partially reversed the BIX01294-mediated inhibition of pro-fibrotic transcripts *ACTA2*, *COL1A1*, *FN1*, and *CTGF* (Fig. 4e-h). We conclude that potent anti-fibrotic effect of a G9a inhibitor can be partially explained by its effects on de-repressing *CEBPA* expression.

### CEBPA expression can be restored by CRISPR activation

**Fig. 5:**
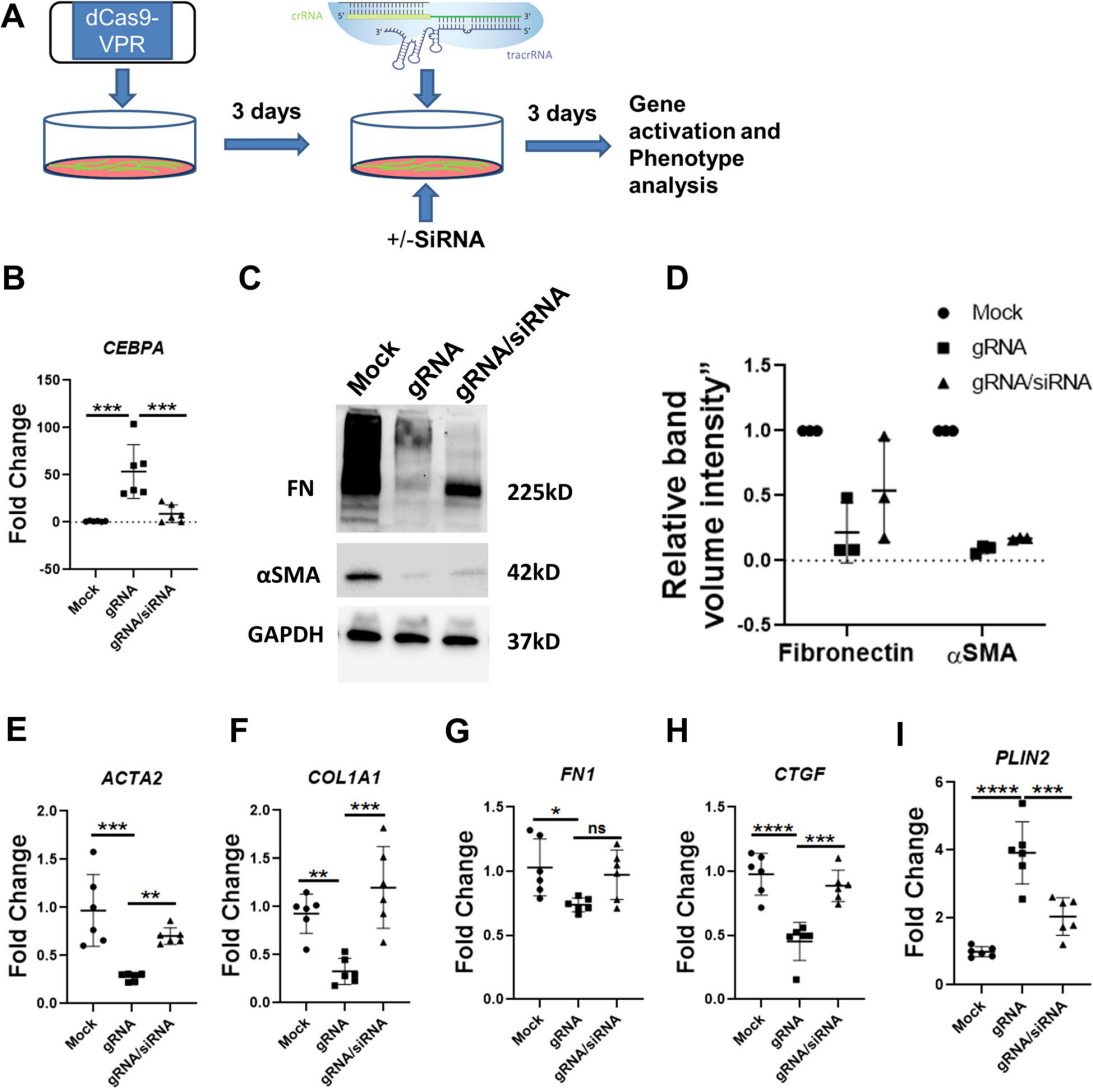
CEBPA expression can be restored by CRISPR activation. **a**) Schematic illustration of the process of CRISPR gene activation (reproduced image is from Horizon Discovery and used with their permission) **b**) qRT-PCR analysis of *CEBPA* expression in the dCAS9 expressing IPF fibroblasts with non-targeting gRNA or CEBPA gRNA or CEBPA gRNA&CEBPA siRNA together for 3 days. **c-d**) Western blot analysis and quantification of Fibronectin and αSMA expression in the dCAS9 expressing IPF fibroblasts with non-targeting gRNA or CEBPA gRNA or CEBPA gRNA&CEBPA siRNA together for 3 days. **e-i**) qRT-PCR analysis showing *ACTA2*, *COL1A1*, *FN1*, *CTGF* and *PLIN2* transcript levels in the dCAS9 expressing IPF fibroblasts with non-targeting gRNA or CEBPA gRNA or CEBPA gRNA&CEBPA siRNA together for 3 days. Data are expressed as mean ± SD (*p < 0.05, ** *p* < 0.01, ***p < 0.001, **** *p* < 0.0001)

Recent CRISPR technology advances have enabled the activation of gene expression without modifying the genome [21, 23, 24]. CRISPR activation (CRISPRa) is a tool that uses a modified version of Cas9 referred to as dCas9. This mutant lacks endonuclease activity. CRISPRa employs dCas9 fused to one of a variety of transcriptional activation domains, which can be directed to promoter regions by gRNAs that recruit additional transcriptional activators to upregulate expression of the target gene [21]. Previous study found that dCas9-VP64, dCas9-p65, and dCas9-Rta showed the most meaningful reporter induction among the hybrid proteins tested [21] and led to the design of an improved tripartite transcriptional activator called VP64-p65-Rta (VPR), fused to nuclease-null Cas9 [25]. Here we tested if CEBPA expression can be regulated by CRISPR activation and mediate effects on fibroblast fate similar to transient transfection with exogenous plasmid. We first transfected the plasmid expressing dCas9-VPR to IPF fibroblasts. After 3 days, synthetic CEBPA guide RNA targeting the transcriptional start site of CEBPA was transfected into the same cells (Fig. 5a). After another 3 days, we found by qRT-PCR that transfection of CEBPA-gRNA increased expression of *CEBPA* mRNA up to 100 fold (Fig. 5b), as well as lipofibroblast marker *PLIN2* (Fig. 5I). At the same time, CEBPA-gRNA transfection significantly reduced the expression of pro-fibrotic genes *ACTA2*, *COL1A1*, *FN1*, and *CTGF* (Fig. 5e-f). Decreased expression of ACTA2 and FN1 were confirmed at the protein level by Western blotting (Fig. 5 c, d). To confirm that the anti-fibrotic effects of our CRISPRa approach are an on-target effect of increased CEBPA expression, we co-transfected *CEBPA* siRNA with CEBPA-gRNA for 3 days. CEBPA siRNAs significantly reduced the expression of CEBPA and increased the expression of pro-fibrotic gene *ACTA2*, *COL1A1*, *FN1*, and *CTGF* (Fig. 5e-f) compared with CEBPA gRNA alone. These results confirm that *CEBPA* expression can be enhanced in a targeted fashion with CRISPR activation, and results in a fibroblast switch away from a pro-fibrotic and toward a lipofibroblast fate.

## Discussion

The accumulation of collagen-secreting activated fibroblasts is a central feature of IPF [1]. In the normal lung, lipofibroblasts are located in the interstitium, near alveolar type II epithelial cells, and are thought to play important roles in alveolar type II epithelial homeostasis and function [7]. Increasing evidence has shown that there is loss of lipofibroblasts in lung fibrosis [26], potentially compromising appropriate epithelial-mesenchymal cell interaction needed to maintain or restore homeostasis in the lungs. El Agha et al. recently implicated lipofibroblasts in myofibroblast generation following injury in murine lungs using lineage tracing, and demonstrated the importance of the reverse myofibroblast to lipofibroblast conversion in lung fibrosis resolution and regeneration [3]. Moreover, they also demonstrated the loss of expression of lipofibroblast-associated genes *ADRP*, *PPARγ* and *CEBPA* in human IPF. These results are consistent with a broader literature that supports important roles for the plasticity of fibroblasts during fibrosis and resolution, including observations that lipogenic conversion might be a common mechanism of fibrosis resolution during tissue repair [27]. Here we have shown that overexpression of CEBPA is able to restore the adipogenesis potential of IPF fibroblasts and increase the expression of lipofibroblast gene expression while simultaneously reducing pro-fibrotic gene and protein expression. Our results also show that CEBPA overexpression antagonizes TGF-β signaling. We speculate that such responses reflect transcriptional modulation of TGF-β regulatory genes by CEBPA during this cellular fate transition, including through candidates previously linked to CEBPA such as *TGFBR2* and *PPARG1* [28, 29]. Our results thus demonstrate that CEBPA expression is capable of promoting a phenotype switch from activated pro-fibrotic fibroblasts to lipofibroblasts in lung fibroblasts. Further efforts to enhance CEBPA expression specifically in fibroblasts may have utility in treating progressive fibrotic diseases such as IPF.

Alterations in cell state are often controlled via epigenetic mechanisms, thus drugs targeting epigenetic mechanisms represent a potential strategy to modulate fibroblast phenotype and gene program. G9a inhibitors have been used in improving human iPSC derivation efficacy [30] and cancer therapeutics [31] via facilitating transcription factor engagement to the genome. Previous work has highlighted the importance of epigenetic modulators G9a and EZH2 in the epigenetic silencing of cyclooxygenase-2 in lung fibroblasts from subjects with IPF [32]. More recently, G9a and CBX5 have been identified as critical epigenetic modulators responsible for repressing *PPARGC1A* gene during TGF-β1 and matrix stiffness mediated fibroblast activation [22]. We similarly find that G9a plays a role in CEBPA repression in lung fibroblasts. Further study identified that repression of peroxisome proliferator activated receptor gamma coactivator 1-alpha (PGC1α), encoded by PPARGC1A, is a key driver of fibroblast metabolic dysfunction, pro-fibrotic and pro-senescence signaling and fibrogenic activation in IPF [33]. Similarly, we report here that CEBPA appear to share similar anti-fibrotic effects when expressed in lung fibroblasts, and that its repression promotes fibroblast activation. Whether these effects of CEBPA and PPARGC1A are directly related remains to be determined.

While epigenetic modulators are potent regulators of cell fate, they have widespread effects and potential cytotoxic limitations that challenge their utility in chronic disease management [34]. A novel aspect of our work is the application of CRISPR activation (CRISPRa) to increase expression of CEBPA in a targeted fashion. CRISPR-Cas9 was originally developed to introduce insertions and deletions into specific DNA sequences, whereas CRISPRa employs an endonuclease-dead Cas9 which binds DNA but is not able to cleave it [21, 25]. Unlike genome editing, CRISPRa does not alter the genomic DNA sequence, highlighting the possibility of applying transcriptional control to manipulate disease conditions. Our work uses a dCas9-VPR construct that promotes assembly of a transcriptional complex [21], combined with guide RNAs that target the complex to the promoter region of CEBPA. We find that this approach dramatically elevates *CEBPA* transcript levels, and attenuates pro-fibrotic fibroblast activation in a CEBPA-dependent fashion (Fig. 5). Our results demonstrate the promising potential of the CRISPRa strategy for restoring gene expression lost in chronic lung diseases, and the capability of such approaches to alter fibroblast fate.

In vivo applications of CRISPRa will require surmounting several challenges. For any nucleic acid based therapeutics, one inherent limitation is off target effects. dCas9 mediate CRISPR activation has the unique capability to enhance expression of target genes without introducing DNA double-strand breaks [35]. This avoids the concern of creating undesired permanent mutations in the genome, which will significantly minimize the impact of off target effects. Another fundamental challenge is the large size of the CRISPRa-dCas9 system, which exceeds the capacity of viral transduction systems when packaged together [36]. Recently, Liao et al. generated a dual-AAV system and showed that co-injection of AAV-Cas9 with an AAV-gRNA was capable of successfully targeting the utrophin gene in vivo, and could ameliorate muscular dystrophy symptoms in mdx mice [37]. Another proof-of-concept study showed that in utero/in vivo CRISPR gene editing is able to correct harmful mutation causes cystic fibrosis, which will be a promising new approach for treating lung diseases before birth [38]. However, whether CEBPA restoration can be achieved in the fibroblasts in vivo with CRISPR activation, and whether its effects would be beneficial remains unknown, representing a limitation of this study. Future work will be needed to test both the feasibility and efficacy of such an approach. Thus, continuing development of CRISPR and other technologies to target CEBPA may pave the way toward in vivo modulation of fibroblasts.

## Conclusion

In summary, our results demonstrate a critical role of CEBPA in modulating fibroblasts between lipogenic and myogenic fates. Our use of CRISPR/dCas9-mediated gene activation serves as proof of concept that this approach can be used to regulate fibroblast fate by targeted transcriptional control. Combined with the prior observation that CEBPA is reduced in the lungs of subjects with IPF [15], and our observation that *CEBPA* expression is specifically reduced in IPF fibroblasts, our results support efforts to specifically restore CEBPA expression in the lung to switch fibroblast state and ultimately arrest or reverse fibrotic ECM deposition.

## Supplementary information

**Additional file 1: Figure S1. a**) Cell number quantification by DAPI in CEBPA knock down (48 h), CEBPA overexpression (48 h) and their control. qRT-PCR analysis showing **b**) SERPINE1 **c**) CDKN2A **d**) GLB1 transcript levels in the C/EBPα-overexpressing IPF fibroblasts compared and empty vector transfected control. Data are expressed as mean ± SD (*p < 0.05, ** *p* < 0.01, ***p < 0.001, **** *p* < 0.0001)

## Data Availability

The authors confirm that the data supporting the findings of this study are available within the article.

## Abbreviations

ACTA2: Smooth muscle alpha (α)-2 actin
Cas9: CRISPR associated protein 9
CDKN2A: Cyclin dependent kinase inhibitor 2A also known as P16.
CEBPA: CCAAT/enhancer-binding protein alpha
COL1A1: Collagen, type I, alpha 1
CRISPR: Clusters of regularly interspaced short palindromic repeats
CTGF: Connective tissue growth factor
dCas9: Cas9 Endonuclease Dead
FN1: Fibronectin 1
GLB1: Galactosidase beta 1
HLF: Human lung fibroblast
IPF: Idiopathic pulmonary fibrosis
PLIN2: Perilipin-2
PPARA: Peroxisome Proliferator Activated Receptor Alpha
PPARG1: Peroxisome Proliferator Activated Receptor Gamma
PPARGC1A: Peroxisome proliferator-activated receptor gamma coactivator 1-alpha
SERPINE1: Serpin family E member 1 also known as Plasminogen activator inhibitor-1 (PAI-1)
