# Supplementary material for: Targeted regulation of fibroblast state by CRISPR-mediated CEBPA expression
Source: Respir Res. 2019 Dec 11;20:281. doi: 10.1186/s12931-019-1253-1 (PMC6907247; doi:10.1186/s12931-019-1253-1)

**Figure S1: A)** Cell number quantification by DAPI in CEBPA knock down (48 hours), CEBPA overexpression (48 hours) and their control. qRT-PCR analysis showing **B)** SERPINE1 **C)** CDKN2A **D)** GLB1 transcript levels in the C/EBPα-overexpressing IPF fibroblasts compared and empty vector transfected control. Data are expressed as mean ± SD (*p<0.05, ** p<0.01, ***p<0.001, **** p<0.0001).


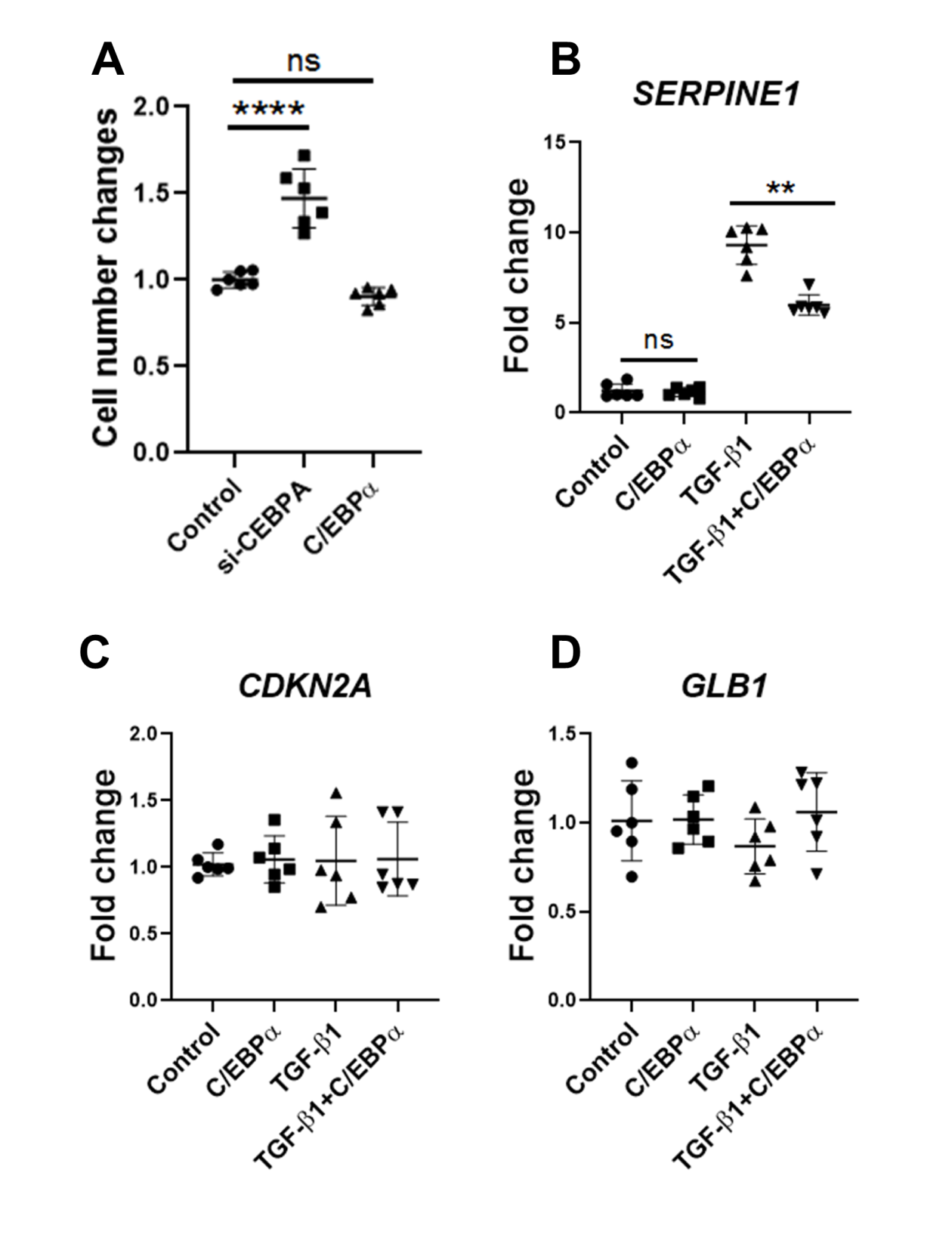

Supplement: Supplementary file 1 — Additional file 1: Figure S1. a) Cell number quantification by DAPI in CEBPA knock down (48 h), CEBPA overexpression (48 h) and their control. qRT-PCR analysis showing b) SERPINE1 c) CDKN2A d) GLB1 transcript levels in the C/EBPα-overexpressing IPF fibroblasts compared and empty vector transfected control. Data are expressed as mean ± SD (*p < 0.05, ** p < 0.01, ***p < 0.001, **** p < 0.0001) [file 12931_2019_1253_MOESM1_ESM.docx]
